# Thyroid Function and All-cause Mortality in the Context of Multimorbidity: Results From 2 Population-based Studies

**DOI:** 10.1210/clinem/dgaf425

**Published:** 2025-07-25

**Authors:** Yanning Xu, Silvan Licher, W Edward Visser, Stephan J L Bakker, Robin P Peeters, Robin P F Dullaart, Layal Chaker

**Affiliations:** Department of Internal Medicine, Erasmus University Medical Center, Rotterdam 3000 CA, The Netherlands; Academic Center for Thyroid Diseases, Erasmus University Medical Center, Rotterdam 3000 CA, The Netherlands; Department of Epidemiology, Erasmus University Medical Center, Rotterdam 3000 CA, The Netherlands; Department of Epidemiology, Erasmus University Medical Center, Rotterdam 3000 CA, The Netherlands; Department of General Practice, Erasmus University Medical Center, Rotterdam 3000 CA, The Netherlands; Department of Internal Medicine, Erasmus University Medical Center, Rotterdam 3000 CA, The Netherlands; Academic Center for Thyroid Diseases, Erasmus University Medical Center, Rotterdam 3000 CA, The Netherlands; Department of Internal Medicine, University Medical Center Groningen, University of Groningen, Groningen 9700 RB, the Netherlands; Department of Internal Medicine, Erasmus University Medical Center, Rotterdam 3000 CA, The Netherlands; Academic Center for Thyroid Diseases, Erasmus University Medical Center, Rotterdam 3000 CA, The Netherlands; Department of Internal Medicine, University Medical Center Groningen, University of Groningen, Groningen 9700 RB, the Netherlands; Department of Internal Medicine, Erasmus University Medical Center, Rotterdam 3000 CA, The Netherlands; Academic Center for Thyroid Diseases, Erasmus University Medical Center, Rotterdam 3000 CA, The Netherlands; Department of Epidemiology, Erasmus University Medical Center, Rotterdam 3000 CA, The Netherlands

**Keywords:** thyroid function, mortality, multimorbidity, free triiodothyronine, free thyroxine

## Abstract

**Background:**

Thyroid dysfunction is common in aging populations and associated with increased noncommunicable disease risk. Complex disease interactions in multimorbidity may influence this association. We aimed to examine the association between thyroid function and all-cause mortality in the context of multimorbidity.

**Methods:**

We included participants with thyroid function measurements and recorded disease status from the PREVEND and the Rotterdam studies and categorized them into 3 groups (no disease, 1 disease, and multimorbidity). We used Cox proportional hazards models for the associations between thyroid function and all-cause mortality. Hazard ratios (HRs) were expressed per 1-unit increment in thyroid function *Z*-scores.

**Results:**

A total of 5537 participants (mean age, 53.0 years) from PREVEND and 9080 participants (mean age, 64.9 years) from the Rotterdam Study were included. Higher free T4 concentrations were associated with a higher all-cause mortality risk in the Rotterdam Study, with HRs per 1-unit increase in a *Z*-score of 1.07 (1.03-1.12), 1.09 (1.04-1.15), 1.21 (1.11-1.31) for individuals with no disease, 1 disease, and multimorbidity, respectively (*P* for trend <.001), whereas a similar but nonsignificant trend was observed in PREVEND. We show a lower mortality risk for higher free T3 concentrations among individuals with 1 disease (HR per 1-unit increase in *Z*-score: 0.82, 0.70-0.97) and multimorbidity (HR, 0.80; 0.61-1.05) (*P* for trend = .002).

**Conclusion:**

We show an association between higher free T4 and mortality for individuals with multimorbidity, whereas lower free T3 was associated with poor survival in individuals with multimorbidity. Our results extend findings from patient populations to people with multimorbidity from the general population. Future research is needed to investigate whether these findings extend to levothyroxine users.

The coexistence of 2 or more chronic illnesses is referred to as multimorbidity. The prevalence of cooccurrence of noncommunicable diseases ranges from 15% to 43%, largely dependent on geographical regions and definitions. Among individuals aged ≥85 years, the prevalence of multimorbidity can reach up to 82% (1). Individuals with multimorbidity have a higher risk of mortality, hospitalization, and extended stay at the hospital (2, 3), causing an increasing burden on the health care system.

Thyroid dysfunction is associated with an increased risk of several major noncommunicable diseases including cardiovascular risk factors and diseases. However, chronic illness can also impact thyroid function. The most common alteration observed is a decrease in T3 without an accompanied elevation in TSH. This phenomenon is referred to as nonthyroidal illness syndrome (NTIS), caused by changes in hypothalamic-pituitary-thyroid axis and thyroid metabolism (4). These alternations have mainly been described in severe illness but evidence indicates that increased disease count and multimorbidity may also be associated with lower free T3 concentrations (5). Although lower free T3 has been associated with long-term prognosis in hospitalized individuals with certain diseases, such as cardiac disease (6) and renal disease (7), its implications in multimorbid individuals from the general population remains largely unknown.

Individuals with multimorbidity might also be more vulnerable to the consequences of thyroid dysfunction. Earlier studies among cardiac and hemodialysis patients indicated that mild thyroid dysfunction as reflected by TSH outside the reference range was associated with worse survival (8, 9). For individuals with multimorbidity, an even more pronounced effect of thyroid dysfunction might be postulated. Given these complexities, different associations between thyroid function and mortality could be implicated among individuals with multimorbidity.

Using data from 2 prospective population-based cohort studies, our objective was to investigate whether the association between thyroid function and all-cause mortality could be modified according to various disease conditions, specifically, no disease, 1 disease, or multimorbidity.

## Methods

### Study Populations

We included data from 2 population-based cohorts from the Netherlands, the Rotterdam Study and the PREVEND cohort, to obtain more comprehensive thyroid function data with free T3 included and enable mutual validation.

The Rotterdam Study included participants aged >40 years old living in the Ommoord district from Rotterdam. Details on the rationale and study design have been described in previous publications (10). The first recruitment started in 1989 and 7983 participants aged >55 years old comprised the subcohort RS-I. Subsequently, in 2000, the second subcohort, RS-II, was initiated and recruited 3011 adults aged >55 years old. The Rotterdam Study was further expanded in 2006 (RS-III) and in 2016 (RS-IV) to include adults aged >45 years and >40 years, respectively. Participants are visited and examined around every 5 years during the follow-up. Thyroid function measurement was performed at the third visit of RS-I and the first visit of RS-II and RS-III, which constituted the baseline for the current study. The Rotterdam Study has been approved by the Medical Ethics Committee of Erasmus MC (registration number MEC 02.1015) and by the Dutch Ministry of Health, Welfare and Sport.

The PREVEND cohort was designed to investigate the risk factors, prevalence and consequences of microalbuminuria in healthy adults in Groningen, the Netherlands. Extensive descriptions of the study design have been published elsewhere (11). From 1997 to 1998, all inhabitants of Groningen aged 28 to 75 were invited to participate. Of 40 856 who responded (47.8% of those invited), those with a urinary albumin excretion (UAE) level ≥ 10 mg/L (n = 7768) and a control group with urinary albumin excretion <10 mg/L (n = 3395) were invited for further study, resulting in 8592 enrolled participants. A second screening started in 2001 including 6894 participants from the original cohort and served as the baseline for this study. The PREVEND cohort was approved by the medical ethics committee of the University Medical Center Groningen (approval number: MEC96/01/022) and all participants provided written informed consent.

**Figure 1.**
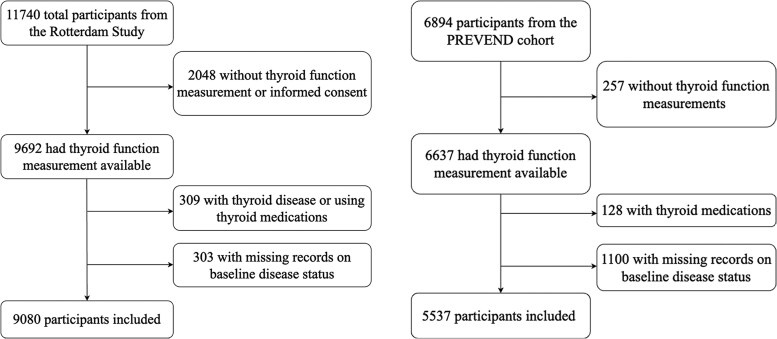
Flowchart of included participants from the Rotterdam Study and the PREVEND cohort.

In both cohorts, we included participants with thyroid function measurements, disease status recorded at baseline, and available vital status during follow-up. Participants taking thyroid medications (levothyroxine, triiodothyronine, antithyroid medication) and those with thyroid diseases were excluded from the current study (Fig. 1).

### Assessment of Thyroid Function

Serum and EDTA anticoagulated plasma samples were stored frozen at −80 °C until analysis. TSH and free T4, and thyroid peroxidase antibodies (TPOAb) were measured by electrochemiluminescence immunoassay ECLIA, Roche in the Rotterdam Study and Roche Modular E170 Analyzer electrochemiluminescent immunoassays (Roche Diagnostics, Mannheim, Germany) in the PREVEND cohort. Reference ranges: TSH 0.4-4.0 mIU/L, free T4 11-25 pmol/L (Rotterdam study); TSH 0.27-4.20 mIU/L, free T4 12-22 pmol/L, free T3 3.1-6.8 pmol/L (PREVEND cohort). The cutoffs to define TPOAb positivity was 35 kU/mL and 34 kIU/L for the Rotterdam and the PREVEND studies, respectively.

### Ascertainment of Disease, Multimorbidity, and All-cause Mortality

Multimorbidity was defined as the presence of 2 or more of the following diseases at baseline: heart disease (including coronary heart disease and heart failure), stroke, chronic respiratory disease (including chronic obstructive pulmonary disease, and asthma), diabetes, and cancer. Our main analysis included 5 diseases, incorporating 7 conditions from the core list of 20 conditions that were recommended for multimorbidity research (12). These conditions were selected because they are the leading contributors to global disability-adjusted life-years (13) and are among the top of the core list of chronic conditions for the measures of multimorbidity. In our sensitivity analyses, we also incorporated 3 additional conditions (depression, dementia, and chronic kidney disease) available in 1 of the cohorts, bringing the total to 10 of 20 conditions in this particular analysis. We did not include the remaining conditions because (1) their prevalence and impact on disability-adjusted life-years are limited and (2) data on these conditions were unavailable or less-well defined. Detailed definitions and determination of each disease are described in Table S1 (14).

Data on all-cause mortality were obtained from municipal records, general practitioners, nursing home, and family in the Rotterdam Study and from municipality records in the PREVEND cohort.

### Assessment of Other Variables in Both Cohorts

Triglycerides, total cholesterol, and high-density lipoprotein (HDL) cholesterol were measured with standard methods in both cohorts (11, 15). Smoking status was defined as current, past, and never. Smoking pack-years were calculated by multiplying the number of cigarette packs smoked per day by the number of years the individual had smoked. Blood pressure was measured twice at the right brachial artery and the mean of both measurements was calculated. Body mass index (BMI) was determined as weight divided by height (kg/m^2^). Use of antihypertensive medication and lipid-lowering medication was obtained by questionnaires. Alcohol consumption was defined as yes or no based on questionnaires.

### Statistical Analysis

Considering the difference between the thyroid function assay used and population heterogeneity between these 2 studies, we standardized the TSH, FT4, and FT3 measurements with *Z*-scores to facilitate the comparison. According to the baseline disease count, we categorized participants into 3 groups: no disease, 1 disease, and multimorbidity (≥2 diseases). Kaplan-Meier survival curves were generated to visualize differences in survival probabilities across thyroid function categories within each group. Cox proportional hazards models were used to investigate the association between thyroid function and all-cause mortality within each group. Hazard ratios (HRs) were expressed per 1 SD increment in thyroid function *Z*-scores. The first model was adjusted for age and sex. The second model further took systolic blood pressure, smoking, alcohol intake, BMI, total cholesterol, HDL cholesterol, triglycerides, and lipid-lowering agents into consideration. We examined the proportional hazard assumption with scaled Schoenfeld residual plot, and no violations of the assumption were identified in this study. We undertook multiple imputations to deal with missing values (maximum missing rate: 18.5% for alcohol consumption) and generated 5 imputed datasets to account for the uncertainty (16).

Sensitivity analyses were conducted to assess the robustness of our results as follows: (1) including only participants with thyroid function within the reference intervals; (2) 1-step analysis that combined data from both cohorts and fit into 1 model; (3) restricting analyses to participants aged >45 years old in the PREVEND cohort to facilitate the comparison with the Rotterdam Study; (4) including chronic kidney disease in the disease count; (5) including depression and neurodegenerative disease (dementia and Parkinsonism) in the disease count only for the Rotterdam Study; (6) including only participants with negative TPOAb; (7) adjusting for smoking pack-years in the second model to account for both smoking duration and intensity; and (8) sequentially excluding individual comorbidities from the multimorbidity definition to explore whether specific conditions were driving the observed associations. All the analyses were conducted with R, version 4.3.2.

## Results

We included 5537 participants from the PREVEND cohort and 9080 participants from the Rotterdam Study. Compared to the PREVEND cohort, a higher prevalence of chronic diseases except for chronic kidney disease was shown in the Rotterdam Study (Fig. S1 (14)). The 3 most common diseases included in the main analysis were diabetes (Rotterdam Study: 12.1%; PREVEND: 6.1%), chronic respiratory disease (Rotterdam Study: 11.1%; PREVEND: 9.4%), and heart disease (Rotterdam Study: 8.9%; PREVEND: 5.5%). Baseline characteristics across participants with different disease status are described in Table 1. As the number of morbidities increased, participants tended to be older and had a higher percentage of men. Thyroid function measurements were similar among groups except for slightly lower free T3 concentrations in participants with multimorbidity. One participant with a single disease and another with multimorbidity had free T3 levels below the reference range with normal TSH. The percentage of participants who were positive for TPOAb was lower in participants with multimorbidity both in the PREVEND and Rotterdam cohort. Participants with multimorbidity at baseline showed higher BMI and systolic blood pressure levels and used antihypertensive medication and lipid-lowering medication more frequently. They had higher triglycerides levels, but lower levels of total cholesterol and HDL cholesterol.

**Table 1.** Baseline characteristics of participants according to baseline disease status

	PREVEND cohort				Rotterdam Study			
	Overall	Baseline disease status			Overall	Baseline disease status		
		No disease	One disease	Multimorbidity		No disease	One disease	Multimorbidity
n	5537	4408	949	180	9080	6075	2358	647
Age, y	53.02 (12.00)	51.45 (11.58)	58.15 (11.71)	64.55 (9.82)	64.92 (9.68)	63.58 (9.26)	66.99 (9.92)	69.99 (9.56)
Women, n (%)	2778 (50.2)	2290 (52.0)	418 (44.0)	70 (38.9)	5050 (55.6)	3588 (59.1)	1218 (51.7)	244 (37.7)
TSH, mIU/L	1.61 [1.12, 2.34]	1.62 [1.13, 2.34]	1.57 [1.08, 2.32]	1.46 [1.01, 2.38]	1.90 [1.29, 2.75]	1.90 [1.31, 2.75]	1.93 [1.28, 2.80]	1.81 [1.22, 2.68]
Free T4, pmol/L	15.59 (2.31)	15.59 (2.28)	15.56 (2.40)	15.63 (2.55)	15.63 (2.24)	15.61 (2.19)	15.63 (2.33)	15.80 (2.41)
Free T3, pmol/L	4.87 (0.62)	4.88 (0.62)	4.86 (0.62)	4.77 (0.56)				
TPOAb positivity, n (%)	584 (10.6)	485 (11.0)	96 (10.1)	11 (6.1)	1096 (12.1)	742 (12.2)	291 (12.3)	63 (9.8)
BMI, kg/m^2^	26.65 (4.33)	26.30 (4.12)	27.75 (4.70)	29.26 (5.48)	27.21 (4.19)	26.87 (3.97)	27.76 (4.50)	28.37 (4.62)
Systolic blood pressure, mm Hg	126.08 (18.66)	124.77 (18.11)	130.17 (19.46)	136.66 (21.17)	139.59 (21.08)	138.27 (20.80)	142.10 (21.45)	142.86 (21.20)
Total cholesterol, mmol/L	5.43 (1.04)	5.44 (1.03)	5.43 (1.07)	5.00 (1.07)	5.72 (1.02)	5.82 (0.98)	5.59 (1.05)	5.27 (1.06)
HDL cholesterol, mmol/L	1.25 (0.31)	1.27 (0.31)	1.18 (0.32)	1.13 (0.28)	1.40 (0.41)	1.44 (0.41)	1.34 (0.40)	1.25 (0.38)
Triglycerides, mmol/L	1.36 (1.02)	1.30 (0.96)	1.56 (1.21)	1.63 (1.20)	1.53 (0.84)	1.47 (0.77)	1.62 (0.90)	1.79 (1.08)
Smoking status, n (%)								
No	1611 (29.1)	1378 (31.3)	208 (21.9)	25 (13.9)	2764 (30.5)	2002 (33.0)	636 (27.0)	126 (19.5)
Past	2364 (42.7)	1809 (41.0)	458 (48.3)	97 (53.9)	4266 (47.0)	2679 (44.1)	1220 (51.8)	367 (56.7)
Current	1562 (28.2)	1221 (27.7)	283 (29.8)	58 (32.2)	2045 (22.5)	1392 (22.9)	499 (21.2)	154 (23.8)
Alcohol consumption, n (%)	4173 (75.4)	3406 (77.3)	649 (68.4)	118 (65.6)	7470 (82.4)	5096 (84.0)	1908 (81.0)	466 (72.1)
Use of antihypertensive medication, n (%)	1146 (20.7)	672 (15.2)	352 (37.1)	122 (67.8)	2944 (32.4)	1472 (24.2)	1053 (44.7)	419 (64.8)
Use of lipid-lowering medication, n (%)	466 (8.4)	215 (4.9)	180 (19.0)	71 (39.4)	1499 (16.5)	725 (11.9)	539 (22.9)	235 (36.3)
Follow-up time, years	14.1 [11.0, 14.7]	14.1 [12.1, 14.8]	13.8 [9.5, 14.5]	11.3 [7.6, 14.0]	14.5 [11.2, 19.4]	14.9 [12.4, 20.1]	13.8 [8.8, 18.1]	10.6 [5.1, 14.9]

Values are presented as mean (SD) or median (interquartile range) for continuous variables and number (percentage) for categorical variables.

Abbreviations: BMI, body mass index; TPOAb, thyroid peroxidase antibodies.

Survival curves for individuals with different thyroid function and baseline disease status are shown in Fig. S2 (14). In Cox proportional hazard models adjusted for relevant confounders, we did not identify an association between TSH concentrations and all-cause mortality regardless of the number of chronic diseases at baseline either in the PREVEND cohort nor the Rotterdam Study (Table 2). Higher FT4 concentrations were associated with a higher risk of all-cause mortality across 3 groups in the Rotterdam Study. The magnitude of effect estimates increased with the increase in number of diseases, with HRs of 1.07 (95% CI, 1.03-1.12), 1.09 (1.04-1.15), and 1.21 (1.11-1.31) for per 1-unit increase in *Z*-score (ie, per SD increase) in free T4 among individuals with no disease, 1 disease, and multimorbidity respectively (*P* for trend <.001). Although the results from the PREVEND cohort did not reach statistical significance, we observed an association between free T4 and all-cause mortality with similar effect estimates across individuals with different concomitant disease status. Higher concentrations of free T3 were associated with a lower risk of all-cause mortality in individuals with 1 disease (HR per 1-unit increase in *Z*-score: 0.82; 95% CI 0.70-0.97) and those with multimorbidity (HR, 0.80; 0.61-1.05) respectively, whereas no association was identified for individuals with no disease (HR, 1.00; 0.90-1.12) (*P* for trend = .002).

**Table 2.** Associations between thyroid function and all-cause mortality among individuals with and without multiple chronic diseases (full range of thyroid function parameters)

	No disease			1 disease			Multimorbidity		
	n/N	Model 1 HR (95% CI)	Model 2 HR (95% CI)	n/N	Model 1 HR (95% CI)	Model 2 HR (95% CI)	n/N	Model 1 HR (95% CI)	Model 2 HR (95% CI)
*PREVEND*									
TSH	440/4408	0.96 (0.88-1.05)	0.98 (0.90-1.07)	271/949	0.99 (0.88-1.11)	1.03 (0.91-1.17)	107/180	0.86 (0.70-1.06)	0.91 (0.74-1.13)
Free T4	440/4406	1.12 (1.02-1.23)	1.08 (0.98-1.18)	271/949	1.09 (0.96-1.23)	1.05 (0.93-1.19)	107/180	1.09 (0.92-1.30)	1.10 (0.91-1.32)
Free T3*^a^*	440/4407	1.02 (0.91-1.14)	1.00 (0.90-1.12)	271/949	0.90 (0.76-1.05)	0.82 (0.70-0.97)	106/179	0.89 (0.70-1.13)	0.80 (0.61-1.05)
*Rotterdam Study*									
TSH	2421/6075	0.96 (0.92-0.99)	0.97 (0.93-1.004)	1417/2358	0.97 (0.93-1.02)	0.98 (0.93-1.02)	512/647	0.96 (0.88-1.04)	0.96 (0.88-1.04)
Free T4*^b^*	2418/6071	1.09 (1.05-1.14)	1.07 (1.03-1.12)	1417/2357	1.11 (1.05-1.17)	1.09 (1.04-1.15)	512/647	1.19 (1.10-1.29)	1.21 (1.11-1.31)

HRs are expressed per 1-unit increment in thyroid function *Z*-scores. Model 1: age, sex; model 2: model 1 + systolic blood pressure, smoking, alcohol intake, BMI, total cholesterol, HDL cholesterol, triglycerides, and lipid-lowering medication.

Abbreviations: BMI, body mass index; HDL, high-density lipoprotein.

^*a*^ *P* for trend = .002.

^*b*^ *P* for trend <.001.

We conducted several sensitivity analyses to test the robustness of our results. First, we restricted the analysis to participants with thyroid function within the reference range. Consistent results were found with even greater effect sizes (Table 3). Second, we performed 1-step analysis to combine data from both cohorts. Because free T3 was only available from the PREVEND cohort, we were only able to conduct the 1-step analysis for FT4 and TSH. One-step analysis revealed consistent results with the cohort-separate analysis (Table 4). Third, because of different inclusion criteria, participants from the PREVEND cohort were younger than those from the Rotterdam Study. Including participants aged >45 years old from the PREVEND cohort did not substantially change the results (Table S2 (14)). Fourth, given the overrepresentation of individuals with albuminuria in the PREVEND cohort, we also performed sensitivity analyses in which we included chronic kidney disease in the disease counting. In these analyses, similar results were found, except for a lower effect estimate for FT4 in individuals with multimorbidity and the association for lower FT3 concentrations was only indicated for individuals with multimorbidity (Table S3 (14)). Fifth, although depression and degenerative disease were not available in the PREVEND cohort, they are among the most common chronic diseases with a high burden in older people and were included in the previous study on multimorbidity in the Rotterdam Study (17). Therefore, we conducted a sensitivity analysis including these 2 diseases in the disease count, which yielded results similar to those of our main analysis (Table S4 (14)). Sixth, we carried out a sensitivity analysis among participants who were TPOAb negative, and the findings remained consistent with our main results (Table S5 (14)). Seventh, sensitivity analyses adjusting for smoking pack-years in the second model generated results similar to those obtained when adjusting for smoking status (Table S6 (14)). Finally, sensitivity analyses removing each comorbidity from the disease counting did not suggest a specific comorbidity that was driving the observed associations (Table S7 (14)).

**Table 3.** Association between thyroid function and all-cause mortality among individuals with and without multiple chronic diseases (TSH, free T4, and free T3 within the reference range)

	No disease			One disease			Multimorbidity		
	n/N	Model 1 HR (95% CI)	Model 2 HR (95% CI)	n/N	Model 1 HR (95% CI)	Model 2 HR (95% CI)	n/N	Model 1 HR (95% CI)	Model 2 HR (95% CI)
*PREVEND*									
TSH	391/3989	0.93 (0.80-1.07)	0.96 (0.82-1.12)	245/845	0.97 (0.81-1.18)	1.10 (0.91-1.33)	92/158	0.89 (0.66-1.21)	0.94 (0.69-1.29)
Free T4	391/3989	1.14 (1.01-1.28)	1.08 (0.96-1.22)	245/845	1.05 (0.90-1.23)	1.01 (0.86-1.18)	92/158	1.11 (0.88-1.41)	1.24 (0.95-1.63)
Free T3*^a^*	391/3989	1.00 (0.86-1.16)	0.96 (0.83-1.12)	245/845	0.91 (0.75-1.10)	0.80 (0.65-0.97)	92/158	0.75 (0.56-1.01)	0.74 (0.55-0.99)
*Rotterdam Study*									
TSH	2154/5451	0.92 (0.87-0.97)	0.92 (0.87-0.97)	1217/2041	0.96 (0.89-1.03)	0.97 (0.90-1.04)	459/581	0.93 (0.82-1.06)	0.92 (0.80-1.05)
Free T4*^b^*	2154/5451	1.10 (1.05-1.15)	1.07 (1.02-1.13)	1217/2041	1.13 (1.06-1.20)	1.11 (1.04-1.18)	459/581	1.21 (1.11-1.33)	1.22 (1.11-1.34)

HRs were expressed per 1-unit increment in thyroid function *Z*-scores. Model 1: age, sex; model 2: model 1 + systolic blood pressure, smoking, alcohol intake, BMI, total cholesterol, HDL cholesterol, triglycerides, and lipid-lowering medication.

Abbreviations: BMI, body mass index; HDL, high-density lipoprotein.

^*a*^ *P* for trend = .01.

^*b*^ *P* for trend <.001.

**Table 4.** Associations between thyroid function and all-cause mortality among individuals with and without multiple chronic diseases combining TSH and free T4 data from the PREVEND study and Rotterdam study (1-step analysis)

	No disease			One disease			Multimorbidity		
	n/N	Model 1 HR (95% CI)	Model 2 HR (95% CI)	n/N	Model 1 HR (95% CI)	Model 2 HR (95% CI)	n/N	Model 1 HR (95% CI)	Model 2 HR (95% CI)
TSH	2861/10 483	0.96 (0.92-0.99)	0.97 (0.94-1.00)	1688/3307	0.98 (0.94-1.02)	0.98 (0.94-1.03)	619/827	0.94 (0.87-1.02)	0.95 (0.88-1.03)
Free T4*^a^*	2858/10 477	1.10 (1.06-1.14)	1.07 (1.03-1.11)	1688/3306	1.11 (1.06-1.16)	1.09 (1.03-1.14)	619/827	1.17 (1.09-1.26)	1.17 (1.09-1.27)

HRs were expressed per 1-unit increment in thyroid function *Z*-scores. Model 1: age, sex; model 2: model 1 + systolic blood pressure, smoking, alcohol intake, BMI, total cholesterol, HDL cholesterol, triglycerides, and lipid-lowering medication.

Abbreviations: BMI, body mass index; HDL, high-density lipoprotein.

^*a*^ *P* for trend <.001.

## Discussion

In the current study encompassing 2 longitudinal cohort studies, we determined that individuals with higher free T4 concentrations had a higher risk of all-cause mortality even in the absence of morbidities, and proportional to the number of chronic diseases they had at baseline. In multivariate analyses, low free T3 concentrations were associated with increased all-cause mortality in individuals with chronic illness, and this association might be stronger among those with multimorbidity. These findings highlight that higher free T4 and low free T3 concentrations are both important determinants for survival among individuals with multimorbidity from the general population. Although similar findings have been described in hospitalized patients, this has not previously been confirmed in the general population in the context of multimorbidity.

Higher free T4 concentrations were associated with a higher incidence of all-cause mortality, which was observed in individuals both with or without chronic disease at baseline. Several studies conducted within the general population have provided evidence of an increased mortality risk with higher free T4 concentrations (18-20). Studies that assessed this association in individuals with disease have been focused on specific diseases. Results of these studies are consistent with our results, showing an association between high freeT4 concentrations and increased risk of mortality or worse prognosis in patients with heart failure (21), coronary heart disease (22, 23), and chronic kidney disease (24). Although few studies have examined the association in multimorbid individuals from the general population, research on hospitalized older patients and intensive care patients provides valuable insights into multimorbidity in more acute and severe settings. In line with our results, De Alfieri et al revealed that high free T4 concentrations were related to long-term mortality in very old patients (mean age 84 years) hospitalized for an acute disease (25). In contrast, studies on intensive care units mostly reported no or negative association of free T4 (26, 27), which is likely attributable to greater severity of the disease and nutrition deprivation. Nutritional deprivation can lower free T4 levels by suppressing the hypothalamic-pituitary-thyroid axis through reduced leptin levels, decreasing TSH and free T4, and altering thyroid hormone metabolism via increased deiodinase 3 activity (4). Studies on individuals with multimorbidity in a nonhospitalized setting are scarce, and our study, conducted in community-dwelling populations, showed an increased mortality risk among multimorbid individuals with higher free T4 concentration. Although the HR of 1.21 per SD increase in free T4 may appear modest, free T4 levels can vary by up to 14 pmol/L within in the reference range. When the effect estimate across the full reference range is extrapolated, it corresponds to an HR of 3.11, suggesting that individuals with free T4 levels at the upper limit have a 3-fold higher risk of all-cause mortality compared to those at the lower limit. This might provide complementary valuable insights for general practitioners and outpatient clinics in identifying individuals at higher risk of poor survival. These findings could also aid in interpreting previous FT4 results in the general population within the context of multimorbidity.

NTIS, characterized by low serum free T3 concentrations, is commonly observed in patients in the intensive care unit and with severe disease status (4). Extensive evidence has accumulated that low free T3 concentrations could be a good indicator of prognosis and long-term clinical outcomes for patients in acute critical conditions but also with chronic illnesses (4, 6). In this study, we did not observe substantial differences in TSH, and free T4 concentrations, but slightly lower concentrations of free T3 among participants with multimorbidity. This is likely explained by the design of our cohort studies, which required visits to each research center, suggesting the inclusion of relatively healthy participants. Our study revealed that relatively low free T3 concentrations, even within the reference range, were associated with a higher risk of mortality in individuals with chronic diseases, particularly in those with multimorbidity. Similar results were also shown in a study conducted by Strich et al, where low T3 was associated with poor survival in the unhealthy participants with 1 or more cardiometabolic morbidities (28). Our study confirmed the predictive role of free T3 in individuals with chronic illness and suggested that it may be even more applicable for individuals with multimorbidity. Combined, these data raise the possibility of a phenomenon of imminent NTIS in the context of multimorbidity within the general population.

Although it seems to be conflicting that both high free T4 and low free T3 concentrations were associated with a higher risk of all-cause mortality, similar results have been reported in previous studies on individuals with chronic diseases (22, 24, 29). Several studies have shown that low free T3 to free T4 ratio is associated with poor survival, indirectly supporting our results (30, 31). Only 20% of T3 is generated from the thyroid itself; the remaining 80% relies on the peripheral conversion of T4 by deiodinases 1 (D1) and deiodinases 2 (D2) with deiodinase 3 (D3) being responsible for inactivation of FT3. During NTIS, decreased D1 activity in the liver and reactivation of D3 in the liver and skeletal muscle lead to decreased serum FT3 (4, 32). However, inconsistent with low serum free T3, free T3 concentrations in other tissues might decrease, remain unchanged, or even increase, as a result of organ-specific changes in transporters, receptors and deiodinases (33). For example, tissue T3 concentrations could remain unchanged in heart and skeletal muscle during NTIS (34). This suggests that high free T4 concentrations could still exert detrimental effects on certain tissues. Future studies are still needed to further elucidate the underlying mechanism.

A strength of our study relies in the inclusion of 2 prospective cohort studies with long follow-up time and detailed examination of potential confounders. The consistency of our findings in main analyses across 2 distinct populations with varying age and disease distributions strengthens the robustness of our results and enhances their generalizability. However, some limitations need to be addressed. First, free T3 measurements were not available in the Rotterdam Study, thus we cannot confirm the results for free T3 in the Rotterdam Study. Future studies are warranted to replicate these findings. Second, repeated thyroid function measures were not available, thus potential changes in thyroid function during follow-up could not be taken into account. This might lead to an underestimation of the associations studied (35). Third, given the observational study design, establishing causal relationships is challenging, particularly concerning low free T3 levels. Further research is required to determine whether low free T3 merely reflects the severity of the underlying disease or represents a maladaptive response that contributes to poor clinical outcomes. In addition, despite adjusting for multiple potential confounders, the possibility of residual or unmeasured confounding, such as by socioeconomic status, cannot be ruled out. However, in the Rotterdam Study, additional adjustment for income, marital status, and education did not materially alter the results. These variables were unavailable in the PREVEND cohort, but the E-values for the association between free T3 and all-cause mortality were 1.55 (1-disease group) and 1.60 (multimorbidity group). For comparison, smoking, a well-established risk factor, had HRs of 1.54 and 1.33, respectively. This suggests that any unmeasured confounder would need to have a stronger effect than smoking to fully account for the observed associations, which is unlikely. Fourth, our study lacked sufficient statistical power to perform further stratified analyses based on age and sex or to examine associations with specific causes of mortality. Last, the 2 cohort studies included were predominantly composed of Whites, which limits the generalizability of our findings.

In conclusion, our findings indicated that high free T4 concentrations were associated with increased mortality risk in both individuals with and without multimorbidity. Among individuals with multimorbidity in the general population, lower free T3 was associated with a higher risk of mortality, which is consistent with previous findings among hospitalized patients. This suggests the NTIS may develop gradually rather than being restricted to severe disease conditions. Future studies are required to explore whether these findings are applicable to levothyroxine users.

## Data Availability

All datasets analyzed during the current study are not publicly available but are available from the corresponding author on reasonable request.
